# The Impact of Integrated Patient Education on Short-Term Revisit Rates in Healthcare Settings: A Quality Improvement Project

**DOI:** 10.7759/cureus.56512

**Published:** 2024-03-19

**Authors:** Abdulla Alqallaf

**Affiliations:** 1 General Practice, International Medical Center, Riffa, BHR

**Keywords:** quasi-experimental study, revisit rates, healthcare outcomes, electronic health records, patient education

## Abstract

Background: Patient education plays a critical role in healthcare, influencing outcomes and resource utilization. However, effectively integrating patient education into clinical practice remains challenging due to time constraints and inconsistencies in information delivery. Enhancements in Electronic Health Records (EHR) offer potential solutions by facilitating customized, quality education delivery. This study investigates the impact of an EHR-enhanced patient education intervention on short-term revisit rates to healthcare facilities.

Methods: A quasi-experimental, pre-test/post-test design without a control group was employed at the International Medical Center in Riffa, Bahrain. The intervention consisted of modifications to the EHR system to support patient education, along with staff training on effective education delivery. Patient revisit rates within seven days post-consultation were compared before and after the intervention using chi-square tests and logistic regression, adjusting for potential confounders.

Results: A total of 1,239 patients participated in the study, which was divided into two groups: 754 patients in the pre-intervention group and 485 patients in the post-intervention group. A significant change was observed in the patient revisit rates: in the pre-intervention group, 53.32% of patients (402 out of 754) returned within seven days, compared to 41.44% of patients (201 out of 485) in the post-intervention group, with a p-value < 0.01.

Conclusion: Enhancements to EHR systems, combined with comprehensive staff education on patient education, can lead to significant reductions in short-term patient revisits. This underscores the importance of integrating technological and educational interventions in healthcare settings to improve patient outcomes and efficiency.

## Introduction

Patient education is pivotal in today’s healthcare landscape, significantly influencing patient outcomes and the efficient use of healthcare resources [1-3]. By equipping patients with the necessary knowledge and skills to manage their health conditions, patient education promotes healthier behaviors, improves medication adherence, and decreases the likelihood of hospital readmissions [4,5]. The correlation between high-quality patient education and improved health outcomes underscores its crucial role in enhancing the quality and sustainability of healthcare systems.

Despite its acknowledged importance, the integration of effective patient education into everyday clinical practice faces notable hurdles [6-8]. Healthcare providers often encounter time constraints during patient consultations, which hampers their ability to offer in-depth, personalized education [9,10]. Furthermore, inconsistencies in the quality and delivery of information across various healthcare settings can create disparities in patient knowledge and outcomes. These challenges highlight the urgent need for innovative approaches that streamline patient education without compromising care quality.

Health Information Technology (HIT), and particularly Electronic Health Records (EHR) systems, emerge as a viable solution for bolstering patient education [6-8]. EHR systems facilitate the seamless integration of patient education into clinical workflows, ensuring uniform, high-quality information delivery tailored to individual health conditions. Furthermore, EHR-based interventions can utilize patient data to customize educational materials, making them more pertinent and engaging [10]. This tailored approach not only improves patients' comprehension of their health conditions but also motivates them to actively participate in their management.

Investigating this research gap is crucial for several reasons. Firstly, understanding the effects of EHR-based patient education on short-term revisit rates can offer insights into the intervention's ability to mitigate immediate post-consultation complications and uncertainties, identifying a potential window for preventing adverse outcomes. Secondly, minimizing unnecessary revisits can significantly relieve the strain on healthcare systems, optimizing resource allocation and improving care quality. Lastly, findings from such research could inform the development of more effective patient education strategies, specifically designed to support patients in the critical days following a consultation.

## Materials and methods

Study design and setting

This quasi-experimental study utilized a pre-test/post-test design without a control group and was conducted at the International Medical Center in Riffa, Bahrain. The setting was chosen for its diverse patient population, making it an ideal environment to evaluate the intervention's broad applicability. The center's average of approximately 750 visits per month underscores the study's potential significance for patient care and clinic operations. The intervention phase began in November 2023, with pre-intervention data collection in October 2023 and post-intervention data collection in December 2023.

Intervention

**Figure 1:**
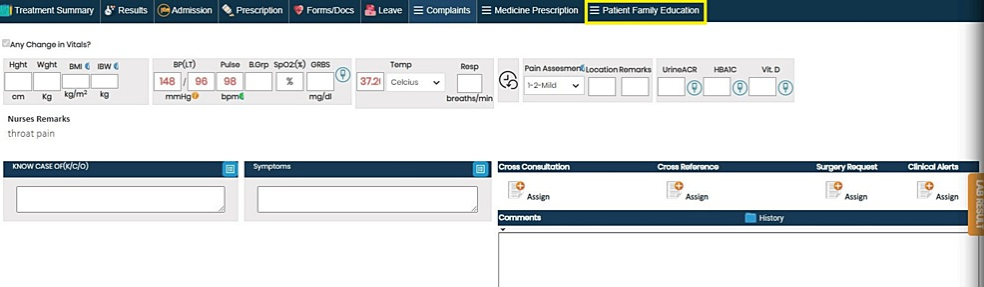
Screenshot from the electronic health record (EHR) highlighting the newly integrated section on patient family education

**Figure 2:**
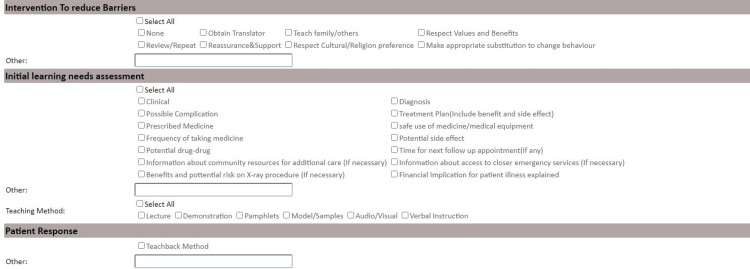
Screenshot from the electronic health record (EHR) showcasing the intervention to reduce barriers and the initial learning needs assessment

**Figure 3:**
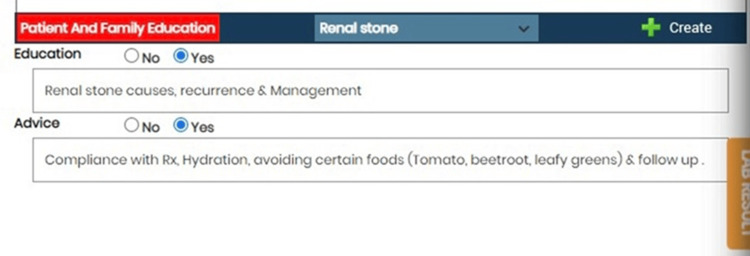
Screenshot from the electronic health record (EHR) displaying an example of patient education documentation

The intervention aimed to enhance patient education through several strategies, including modifications to the EHR system. These modifications involved adding checkboxes to indicate whether patient education was provided during consultations and fields for documenting assessment and education plans. This also included addressing barriers to patient education, such as language barriers, and incorporating standardized educational materials tailored to patient-specific conditions and needs. This was intended to integrate patient education seamlessly into routine care. Staff were trained on these new features through hands-on workshops, with ongoing monitoring to ensure consistent application. Additionally, all clinical staff attended mandatory educational sessions on the importance of patient education, effective communication strategies, and how to utilize the new EHR system features. These sessions were designed to equip staff with the necessary skills for delivering consistent and comprehensive patient education (Figures 1-3).

Participants

The study included all patients visiting the clinics for consultations during the study periods, with broad inclusion criteria to encompass all visit types, ages, and genders. This approach aimed to ensure a comprehensive evaluation of the intervention's impact.

Data collection

Data were retrospectively collected from the hospital's electronic health records, including details of all visits, patient age, gender, the primary reason for the visit, and whether the visit was a primary consultation or a revisit. A revisit was defined as any return to the clinic within seven days of the initial consultation for a related issue. The effectiveness of patient education was inferred from documented patient understanding and compliance, as recorded by treating clinicians in the EHR.

Statistical analysis

The rate of patient revisits before and after the intervention was compared using chi-square tests for categorical data. A p-value of less than 0.05 was considered statistically significant. All statistical analyses were conducted using SPSS software version 26 (IBM Corp., Armonk, NY, USA).

Ethical considerations

Ethical approval was obtained from the appropriate institutional review board of the International Medical Center (Approval Number: IMC/PFEP/PFE/PE3-001), in line with the ethical standards of the 1964 Helsinki Declaration and its later amendments. Patient confidentiality was safeguarded by de-identifying all collected data, and consent for using the data was obtained by the center's guidelines.

## Results

Participant demographics and baseline characteristics

A total of 1,239 patients participated in the study, divided into two groups: 754 patients in the pre-intervention group and 485 patients in the post-intervention group. Table 1 presents the demographics of patients before and after the intervention, including age groups, gender, and nationality.

**Table 1:** Patient demographics before and after intervention The data in the table is represented as N (number of participants) and % (percentage of participants within each category).

Variables	Categories	Pre-Intervention (N=754)		Post-Intervention (N=485)	
		n	%	n	%
Age Group	Under 18 years	474	62.86%	365	75.26%
	18-24 years	104	13.79%	101	20.82%
	25-44 years	10	1.33%	17	3.51%
	45-64 years	89	11.80%	1	0.21%
	65 years and above	77	10.21%	1	0.21%
Gender	Male	455	60.34%	266	54.85%
	Female	299	39.66%	219	45.15%
Nationality	Bahraini	385	51.06%	229	47.22%
	Non-Bahraini	369	48.94%	256	52.78%

Intervention implementation

The implementation of the educational intervention across various clinical departments achieved a 100% staff attendance rate at educational sessions. This indicates strong compliance with the new documentation requirements and practices introduced by the intervention.

Patient revisit rates

**Figure 4:**
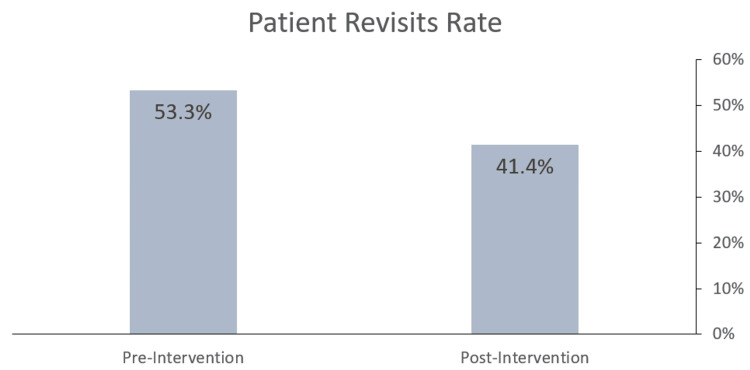
Bar chart showing the patient revisit rate pre- and post-implementation of improvements. The difference is statistically significant (P < 0.01).

A significant change was observed in patient revisit rates: 53.32% of patients (402 out of 754) in the pre-intervention group returned within seven days, compared to 41.44% of patients (201 out of 485) in the post-intervention group. This reduction is statistically significant, underscoring the effectiveness of the intervention in decreasing the frequency of patient revisits (p < 0.01) (Figure 4).

## Discussion

This study aimed to assess the impact of an intervention combining EHR enhancements with staff education on patient education, with a particular focus on its potential to reduce short-term patient revisits. The findings suggest a reduction in the rate of revisits within seven days post-consultation, indicating that the intervention may help in better equipping patients to manage their health, potentially decreasing the need for immediate follow-up care.

The observed decrease in patient revisit rates suggests that integrating patient education more deeply into the clinical workflow, supported by EHR enhancements and staff training, might contribute to improved patient self-management and understanding. This could lead to fewer uncertainties that necessitate rapid follow-up. While these results are encouraging, they echo the broader recognition in healthcare literature of the value of informed patients and the complex interplay of factors that contribute to patient outcomes [3-6].

Prior studies have consistently highlighted the positive effects of patient education on various health outcomes, including medication adherence, self-management of chronic conditions, and overall patient satisfaction [10-15]. However, our study adds to this body of work by examining how technological modifications to EHR systems, in conjunction with staff education initiatives, can facilitate the delivery of patient education. This dual approach appears to reinforce the importance of not only providing information but doing so in a way that is integrated into the healthcare delivery process.

This study, while offering insights into the potential benefits of combining EHR enhancements with staff education on patient education, is not without limitations. The absence of a control group and the reliance on data from a single center may limit the generalizability of the findings. Moreover, measuring the impact solely based on the rate of patient revisits may not capture the full spectrum of benefits associated with improved patient education, such as enhanced quality of life or long-term health outcomes. Future research could benefit from a more robust study design, such as randomized controlled trials, to better isolate the effects of the intervention from other variables. Exploring the long-term impact of these interventions on patient health outcomes and healthcare utilization, as well as their cost-effectiveness, would also be valuable. Additionally, investigating how such interventions can be adapted to various healthcare settings and patient populations could help in tailoring patient education efforts more effectively.

## Conclusions

In conclusion, this study provides preliminary evidence that a targeted intervention, combining enhancements to EHR with comprehensive staff education on patient education, may contribute to a reduction in short-term patient revisits. This suggests a promising avenue for healthcare providers and administrators to improve patient outcomes, enhance efficiency in healthcare delivery, and possibly reduce healthcare costs through the strategic integration of technology and educational initiatives. While the results are encouraging, they underscore the need for further research to explore the scalability, long-term impact, and cost-effectiveness of such interventions across different healthcare settings and populations. Ultimately, this study reinforces the critical role of patient education in the healthcare continuum and the potential of technological innovations to amplify its benefits.
